# Development of a portable leaf photosynthesis and volatile organic compounds emission system

**DOI:** 10.1016/j.mex.2020.100880

**Published:** 2020-04-15

**Authors:** Kolby J. Jardine, Raquel F. Zorzanelli, Bruno O. Gimenez, Emily Robles, Luani Rosa de Oliveira Piva

**Affiliations:** a Earth and Environmental Science Area, Lawrence Berkeley National Laboratory, One Cyclotron Rd, building 84-155, Berkeley, CA 94720, USA; b National Institute for Amazon Research, Department of Forest Management, Ave. Andre Araujo 2936, Manaus, AM 69.080-97, Brazil; c Federal University of Espírito Santo, Ave. Governador Lindemberg, n° 316, Centro, Jerônimo Monteiro, ES 29.550-000, Brazil; d College of Natural Resources, University of California Berkeley, 260 Mulford Hall, Berkeley, CA, 94720, USA; e Federal University of Paraná, Department of Forest Sciences, Ave. Prefeito Lothário Meissner 632, Curitiba, PR 80210-170, Brazil

**Keywords:** VOCs, Photosynthesis, Tropical forest, Light, Leaf, Banana plant

## Abstract

Understanding how plant carbon metabolism responds to environmental variables such as light is central to understanding ecosystem carbon cycling and the production of food, biofuels, and biomaterials. Here, we couple a portable leaf photosynthesis system to an autosampler for volatile organic compounds (VOCs) to enable field observations of net photosynthesis simultaneously with emissions of VOCs as a function of light. Following sample collection, VOCs are analyzed using automated thermal desorption-gas chromatograph-mass spectrometry (TD-GC–MS). An example is presented from a banana plant in the central Amazon with a focus on the response of photosynthesis and the emissions of eight individual monoterpenes to light intensity. Our observations reveal that banana leaf emissions represent a 1.1 +/- 0.1% loss of photosynthesis by carbon. Monoterpene emissions from banana are dominated by trans-β-ocimene, which accounts for up to 57% of total monoterpene emissions at high light. We conclude that the developed system is ideal for the identification and quantification of VOC emissions from leaves in parallel with CO2 and water fluxes.The system therefore permits the analysis of biological and environmental sensitivities of carbon metabolism in leaves in remote field locations, resulting in the emission of hydrocarbons to the atmosphere.

• A field-portable system is developed for the identification and quantification of VOCs from leaves in parallel with leaf physiological measurements including photosynthesis and transpiration.
• The system will enable the characterization of carbon and energy allocation to the biosynthesis and emission of VOCs linked with photosynthesis (e.g. isoprene and monoterpenes) and their biological and environmental sensitivities (e.g. light, temperature, CO_2_).
• Allow the development of more accurate mechanistic global VOC emission models linked with photosynthesis, improving our ability to predict how forests will respond to climate change. It is our hope that the presented system will contribute with critical data towards these goals across Earth's diverse tropical forests.

## Graphical abstract

**Figure:**
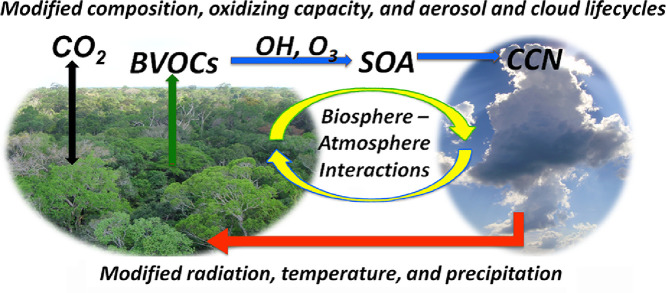

**Table:** Specifications Table

Subject Area	Environmental Science
More specific subject area	Biological and environmental sensitives of photosynthesis and carbon/energy allocation to volatile isoprenoid emissions
Method name	Portable leaf photosynthesis and speciated volatile organic compounds emission system
Name and reference of original method	n/a
Resource availability	Modified portable leaf photosynthesis system (LI-6400XT, LI-COR Inc., USA); thermal desorption (TD) tubes filled with quartz wool, Tenax TA, and Carboxeen 1003 adsorbents (Markes International, UK); portable thermal desorption tube autosampler (Less-P, Signature Science LLC., USA); TD100 automated thermal desorption system (Markes International, UK); gas chromatograph-mass spectrometer (GC–MS, 5975C series, Agilent Technologies, USA).

## Method details

### Background

Emissions of volatile organic compounds (VOCs) from plants represent a few percent of the terrestrial carbon cycle under optimal conditions for photosynthesis, but this percentage can greatly increase during environmental extremes which suppress net primary productivity while stimulating some classes of VOC emissions (e.g. volatile isoprenoids) [1]. Once in the troposphere, VOCs fuel photochemical reactions on local, regional and global scales with impacts on processes that influence both air quality and climate (e.g. production/consumption of ozone and aerosols/cloud interactions) [2]. Of the wide number of environmental factors known to affect photosynthesis and VOC emissions, light, is of fundamental importance [3]. Photosynthetically active radiation (PAR) is directly absorbed by chlorophyll in light harvesting complexes, providing energy for the light reactions of photosynthesis [4]. In forests, light intensity received by leaves depends on a number of factors including leaf area index, height in the canopy, time of day, season, and topography of the landscape in addition to meteorological variables like wind speed and the presence of aerosols and clouds [5].

A number of leaf VOC emissions like isoprene (C_5_H_8_) and monoterpenes (C_10_H_16_) have been shown to be completely dependent on light via photosynthesis, deriving carbon and energy from the light photochemical and dark CO_2_ assimilation reactions, respectively [6]. Thus, studies have used ^13^CO_2_ labeling studies to demonstrate that most of the carbon required for isoprene and monoterpenes from tropical species can come from recent photosynthesis [7]. In addition, a number of studies have provided evidence that isoprene and monoterpenes protect photosynthesis from stress such as high temperature, possibly through a membrane stabilization [8] and antioxidant [9] mechanisms. Thus, there is a tight connection between VOCs like isoprene and monoterpenes and photosynthesis. They are produced from carbon directly derived from the dark reactions of photosynthesis and they may help maintain and protect the light reactions and the operation of the dark reactions. Largely due to the extreme logistical difficulties of studying remote forests in the tropics, very little is known about the relationships between photosynthesis and VOC emissions in these hyper-diverse ecosystems. However, global VOC emission models estimate that the majority of VOCs emitted into the global atmosphere occur in the tropics [10]. Thus, VOCs play important roles in plant physiology, atmospheric chemistry, and terrestrial cycling of carbon and water. However, given the hyper-diversity and remoteness of tropical ecosystems, an outstanding question remains as to which plants emit which compounds, the magnitude of these emissions, and details of mechanistic connections with carbon assimilation processes as a function of environmental variables. While important contributions at the leaf level have been recently described, new studies are needed that focus on characterizing VOC emissions linked with photosynthesis across hyper-diverse ecosystems in the tropics.

To study the relationship between leaf VOC emissions, photosynthesis, and light in a remote primary rainforest, it is imperative that measurements be made on intact leaves in the field, especially in highly productive regions such as the tropics. However, extreme logistical difficulties in the field make current methods challenging, especially in remote areas such as the Amazon rainforest where instrument power requirements, weight, size, and humidity tolerance are limiting factors for VOC emission studies. While limited efforts to quantify the VOC concentrations/emissions from tropical forests have been explored in the tropics, including vertical atmospheric gradients throughout and above the forest canopy, above canopy eddy covariance flux measurements, and remotely piloted drone systems, the lack of methods focused on leaf-level still persists. Although portable leaf photosynthesis systems exist, online spectrometers such as proton transfer reaction - mass spectrometry (PTR-MS) and gas chromatography – mass spectrometry (GC–MS) are extremely difficult to transport and maintain in the field with high energy requirements. Nonetheless, coupling of a VOC system that can identify and quantify VOCs from photosynthesis systems in the ppt-ppb range is ideal because it can allow the evaluation of the dependence of leaf photosynthesis and VOC emissions on environmental conditions such as light, CO_2_, O_3_, and temperature [11]. Unfortunately, a portable system able to identify and quantify VOCs in low concentrations (low ppt-ppb) in dynamic leaf chambers, which is programmable and able to collect time series, and can be taken to the field due to its low weight, small waterproof case, and internal battery does not exist. Here, we present a system with these properties capable of automatically collecting VOC emission time series in parallel with leaf photosynthesis measurements as a function of light intensity. This newly developed system aims to promote studies aiming to understand the functional roles of VOCs in plants and the response of tropical forests to climate variables through quantification of leaf VOC emission/photosynthesis relationship, and studies aiming to characterize how the identities and magnitude of VOC emissions vary across species in hyper-diverse and remote tropical ecosystems. This information will assist in the development of mechanistic photosynthesis and VOC emissions models from tropical ecosystems linked to terrestrial photosynthesis and therefore aid in model predictions of how forests will respond to climate change in the future. It is our hope that using the presented system, future studies will contribute critical data towards these goals across Earth's vast and diverse tropical forests.

### Volatile isoprenoid emissions and net photosynthesis

**Fig. 1:**
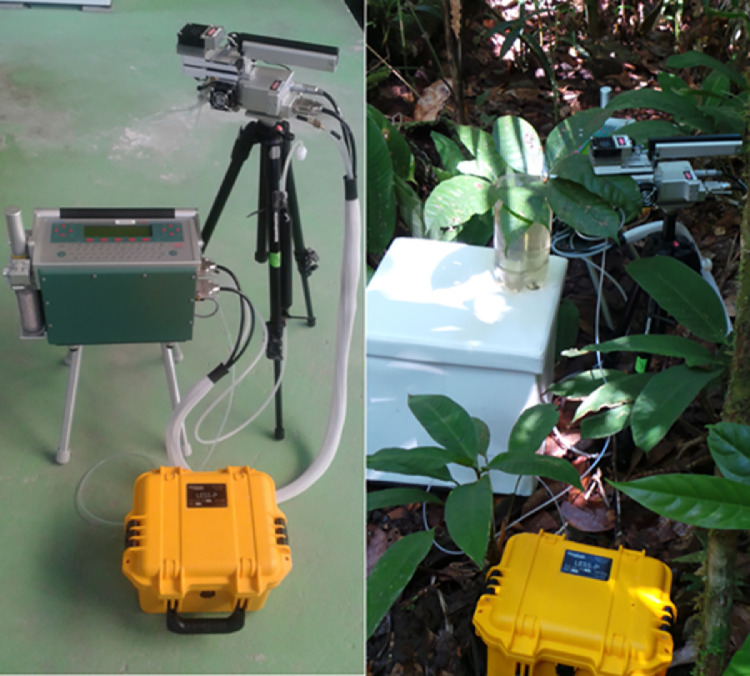
Schematic image of LI6400XT and Less-P coupled system.

For all leaf samples studied for volatile isoprenoid emissions, branch cuttings were conducted in the upper canopy with sun exposed leaves with the assistance of a tree climber utilizing a pole pruner or directly accessed from flux towers. Large branches were removed from the upper canopy (up to 0.5–1.0 m in length) and rapidly recut on the ground under water to maintain the transpiration stream. Net photosynthesis and isoprene and monoterpene emission rates were quantified from leaves during controlled changes in photosynthetically active radiation (PAR) using a commercial leaf photosynthesis system (LI-6400XT, LI-COR Inc., USA) interfaced with a gas chromatograph-mass spectrometer (GC–MS, 5975C series, Agilent Technologies, USA). A modification to the LI-6400XT was made such that a fraction of the air exiting the leaf chamber was diverted to thermal desorption (TD) tubes (Less-P system) for the quantitative collection of any isoprene and monoterpenes emitted from the sample leaf into the chamber (Fig. 1).

TD tubes were purchased commercially, filled with quartz wool, Tenax TA, and Carboxeen 1003 adsorbents (Markes International, UK). All tubing and fittings employed downstream of the leaf chamber were constructed with PFA Teflon (Cole Parmer, USA). Hydrocarbon free ambient air was delivered to the LI-6400XT gas inlet using a capillary-grade hydrocarbon trap (Restek, USA). For all samples, the flow rate of air into the leaf chamber was maintained at 537 ml min^−1^, the internal fan was set to the maximum speed, the leaf temperature was maintained at 30 °C, and the reference CO_2_ concentration entering the chamber was maintained at 400 ppm. Using a tee fitting, air exiting the leaf chamber was delivered to the TD tube (75 ml min^−1^ when collecting) with the remainder of the flow diverted to the vent/match valve within the LI-6400XT. The excess flow entering the vent/match valve was maintained to at least 200 ml min^−1^ by loosely tightening the chamber onto the leaf using the tightening nut.

VOCs exiting the leaf chamber were collected on TD tubes for 10 min at 75 ml min^−1^ automatically during light-response curves using a portable 28 tube auto sampler (Less-P, Signature Science LLC., Austin, TX, USA). The sample leaf was placed in the dark chamber (0 μmol m ^−^ ^2^ s^−1^ PAR), and following a 10 min period of equilibration, the sample and reference infrared gas analyzes were matched, and light curve autoprograms on both LI-6400XT and Less-P were initiated. For the LI-6400XT, the light curve autoprogram consisted of logging data every 30 s while controlling PAR for 10 min at each PAR level (0, 100, 250, 500, 1000, 2000 μmol m^−2^ s^−1^). The autoprogram for the Less-P controlled the sequential sampling of VOCs onto 6 TD tubes, one for each PAR level. An analysis of isoprene and monoterpene concentrations from an empty chamber revealed negligible to undetectable backgrounds. Moreover, leaf isoprene and monoterpene emissions in the dark (PAR flux of 0 μmol m^−2^ s^−1^) also showed negligible to undetectable values. Once collected, the TD tubes were analyzed for isoprene and monoterpene concentrations within 1–5 days using an automated Thermal Desorption – Gas Chromatography – Mass Spectrometry (TD-GC–MS) as described below. Isoprene and monoterpene fluxes were calculated as previously described based on the flow rate of the air into the chamber, the concentration of volatile isoprenoids inside the chamber, and the leaf area inside the chamber (6 cm^2^) [12,13]. The uncertainty in emission measurements are dominated by uncertainties in the volatile isoprenoid gas-phase concentration measurements, as the other parameters used to calculate the emissions (gas flow rate through the leaf chamber and leaf area) are well characterized by the LI-6400XT portable photosynthesis system. We estimate this uncertainty as 20% which includes uncertainty in the calibration of the TD-GC–MS system to gas-phase standards of monoterpenes prepared using the dynamic solution injection technique. This includes uncertainty of the primary liquid standard of monoterpenes in methanol reported by the commercial supplier (Restek, Corp., USA), and uncertainty associated with the evaporative dilution of the primary liquid standard into the gas phase. It should be noted that although leaf temperature was held constant at 30 °C, the absolute and relative humidity (and therefore VPD) changed throughout the light response curve due to the increase in leaf transpiration as stomatal conductance increased from darkness to full light intensity.

### Thermal desorption gas chromatography-mass spectrometry (GC–MS)

Following collection of volatile isoprenoids from leaf emissions, TD tube samples were returned to the analytical laboratory in Manaus, Brazil and analyzed for monoterpenes within two days using TD-GC–MS. TD tubes were analyzed for isoprene and monoterpenes using a thermal desorption system (TD-100, Markes International) interfaced with a gas chromatograph/electron impact mass spectrometer with a triple-axis detector (5975C series, Agilent Technologies, Santa Clara, CA, USA) at INPA, Manaus,

Brazil, as previously described [12]. After loading a tube in the TD-GC–MS system (up to 50 analyzed sequentially), the collected samples were dried by purging for 4 min with 50 ml min^−1^ of ultra-high purity helium (all flow vented out of the split vent) before being transferred (290 °C for 5 min with 50 mfl min^−1^ of helium) to the TD-100 cold trap (air toxics) held at 20 °C. During GC injection, the trap was heated to 290 °C for 3 min while back-flushing with carrier gas at a flow of 6.0 ml min^−1^. Simultaneously, 4.0 ml min^−1^ of this flow was directed to the split and 2.0 ml min^−1^ was directed to the column (Agilent DB624 60 m x 0.32 mm x 1.8 μm). The oven temperature was programmed with an initial hold of 3 min at 40 °C followed by an increase to 230 °C at 6 °C min^−1^. The mass spectrometer was configured for trace analysis with a 15 times detector gain factor and operated in scan mode (*m/z* 35–150).

The GC–MS was calibrated to authentic monoterpene standards (99%, Sigma Aldrich, St. Louis, MO, USA) in methanol using the dynamic solution injection (DSI) technique [14] by dynamic dilution with a hydrocarbon free air flow of 1.0 L min^−1^. Identification of individual monoterpenes from tube samples was performed by comparison of mass spectra with the U.S. National Institute of Standards and Technology (NIST) mass spectral library and by comparison of mass spectra and retention time with the authentic liquid standard which consisted of 10 μg/ml each of the following monoterpenes in methanol [α-pinene (CAS# 80–56–8), camphene (CAS# 79–92–5), d-limonene (CAS# 138–86–3), sabinene (CAS# 3387–41–5), 3-carene (#13,466–78–9), myrcene (CAS# 123–35–3), terpinolene (CAS# 586–62–9), and trans-β-ocimene (CAS# 13,877–91–3)]. Isoprene was calibrated by dynamic dilution of a 1.0 ppm primary standard in nitrogen. TD-GC–MS calibrations were conducted to establish retention times and identities of sample monoterpenes, with peak area responses demonstrated to be highly linear [12].

## Method validation

### Operation of LI6400XT and Less-P system in the field

In the laboratory, the Less-P system was opened, the manifold removed and 28 clean thermal desorption tubes were loaded into the system and fitted with o-rings, with the position and serial number of each tube noted. Once transported to the field site, the tubing connections between the LI6400XT and Less-P instruments were made and the initial environmental conditions were established in the LI6400XT (400 ppm reference CO_2_, 30 ⁰C, 400 μmol *s* ^−^ ^1^, PAR of 0.0 μmol m^−2^ s^−1^). Once the system was running, it was allowed to stabilize for 5 min and a log file was opened and the IRGAs were matched. Following this, the leaf to be sampled was positioned in the LI6400XT leaf chamber allowed to equilibrate in the dark for several minutes. The tightening nut on the leaf chamber was loosely secured to ensure sufficient excess flow exiting the leaf chamber for the VOC sampling (maintained to at least 200 ml min^−1^ excess). The Less-P was then programmed, so that the start time was identical to the start time of the LI6400XT autoprogram, which generates the light curve. Thus, both instruments were set to begin collecting data together without delay of one instrument relative to the other. The two instruments, initiated simultaneously, completed the light curve in 60 min, with one thermal desorption tube collected for 10 min during each of the 6 light levels (totaling 6 tubes for the light curve).

**Fig. 2:**
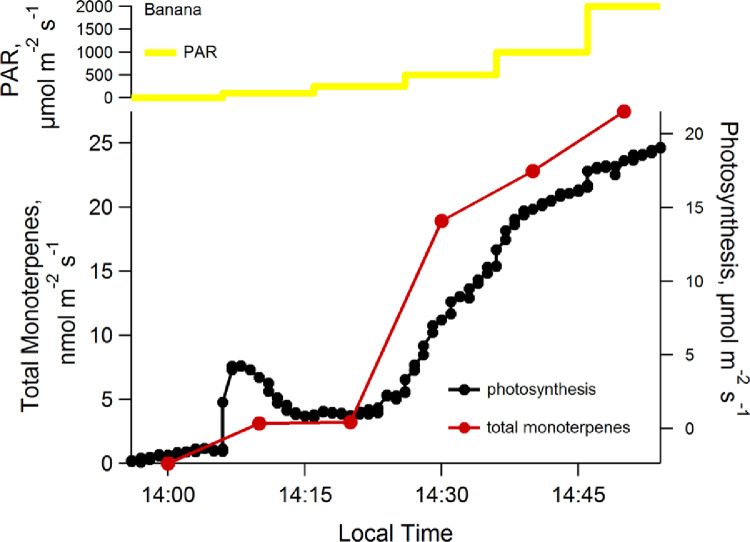
Time series plot showing the response of photosynthesis and the emission rate of the sum of seven monoterpenes to PAR in a banana leaf.

**Fig. 3:**
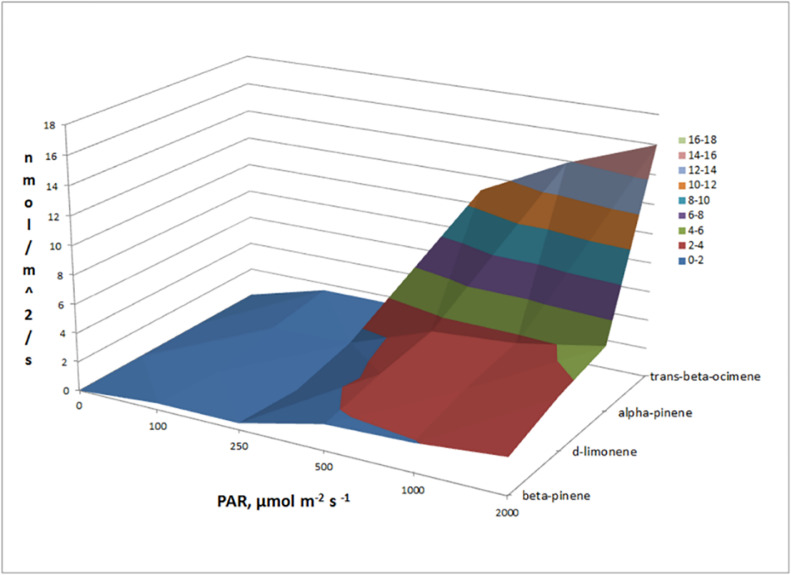
Dependence of the four most abundant monoterpene emissions (trans-β-ocimene, α-pinene, D-limonene and β-pinene) on PAR intensity from a banana leaf.

The system was then tested on the campus of the National Institute for Amazon Research (INPA) in Manaus, Brazil on banana leaves (Figs. 2 and 3). The results of these tests reveal that the developed method has several advantages. The weight and size of the VOC autosampling instrument (Less-P) is low and the system is water proof and able to run continuously for 48 h on a single battery charge. As samples are generally only collected during the daytime, this means that more than four days of continuous data can be collected before recharging the battery. This carrying handle also makes it easy to transport in the field. Moreover, when the analysis of the collected thermal desorption tubes was conducted by GC–MS in the Manaus, Brazil laboratory, extremely low detection limits (less than 100 ppt) for many VOCs could be obtained. Thus, this system combines the advanced capabilities of a high sensitivity laboratory GC–MS with automated sampling in the field during leaf level studies.

### Thermal desorption GC–MS analysis of Less-P tubes

After returning from the field, TD tubes were analyzed in an automated TD-GC–MS system as described in the methods. For this, the TD tubes were removed from Less-P manifold and placed in an appropriate tray in TD-GC–MS. Each tube sample requires roughly 50 min for analysis allowing a full light curve to be completed in 5 h. The concentrations in ppb for each monoterpene found in the sample was then calculated based on the most recent calibration. Emission rates of each monoterpene were then calculated based on the concentration, the leaf area, and the flow rate of air entering the leaf chamber.

### Light dependence of photosynthesis and monoterpenes emissions of a banana leaf

Using a light curve program for LI6400XT an analysis of the light dependence of photosynthesis and VOC emissions was conducted on a banana plant leaf (*Musa* sp.) on July 5, 2014, during a sunny day on the Campus V8 of INPA (National Institute of Research) in Manaus Brazil (Fig. 2). In the beginning of the light curve, the photosynthetic activity in the banana leaf showed negatives values, due to the activity of respiration and other biochemical processes which produce CO_2_ (PAR: 0 µmol m^−2^ s^−1^, net photosynthesis: −1.57 µmol m^−2^ s^−1^). In the dark, total monoterpene emissions were below the detection limits. At the first light level, net photosynthesis became positive and monoterpene emissions were clearly stimulated (PAR: 100 µmol m^−2^ s^−1^, photosynthesis: 1.0 µmol m^−2^ s^−1^; total monoterpene emissions 3.1 nmol m^−2^ s^−1^). As PAR intensities were increased, both net photosynthesis and total monoterpene emission rates were further stimulated up to the highest PAR intensity studied (PAR: 2000 µmol m^−2^ s^−1^, photosynthesis: 19.1 µmol m^−2^ s^−1^; total monoterpene emissions 3.1 nmol m^−2^ s^−1^). While the PAR sensitivity of net photosynthesis and total monoterpene emissions versus light decreased some at high PAR fluxes, light saturation was not observed. When the total monoterpene emission rate was regressed against net photosynthesis rates, a strong linear correlation was observed (R^2^ = 0.94) with 1.39 +/- 0.17% of carbon from net photosynthesis emitted to the atmosphere in the form of monoterpenes.

**Fig. 4:**
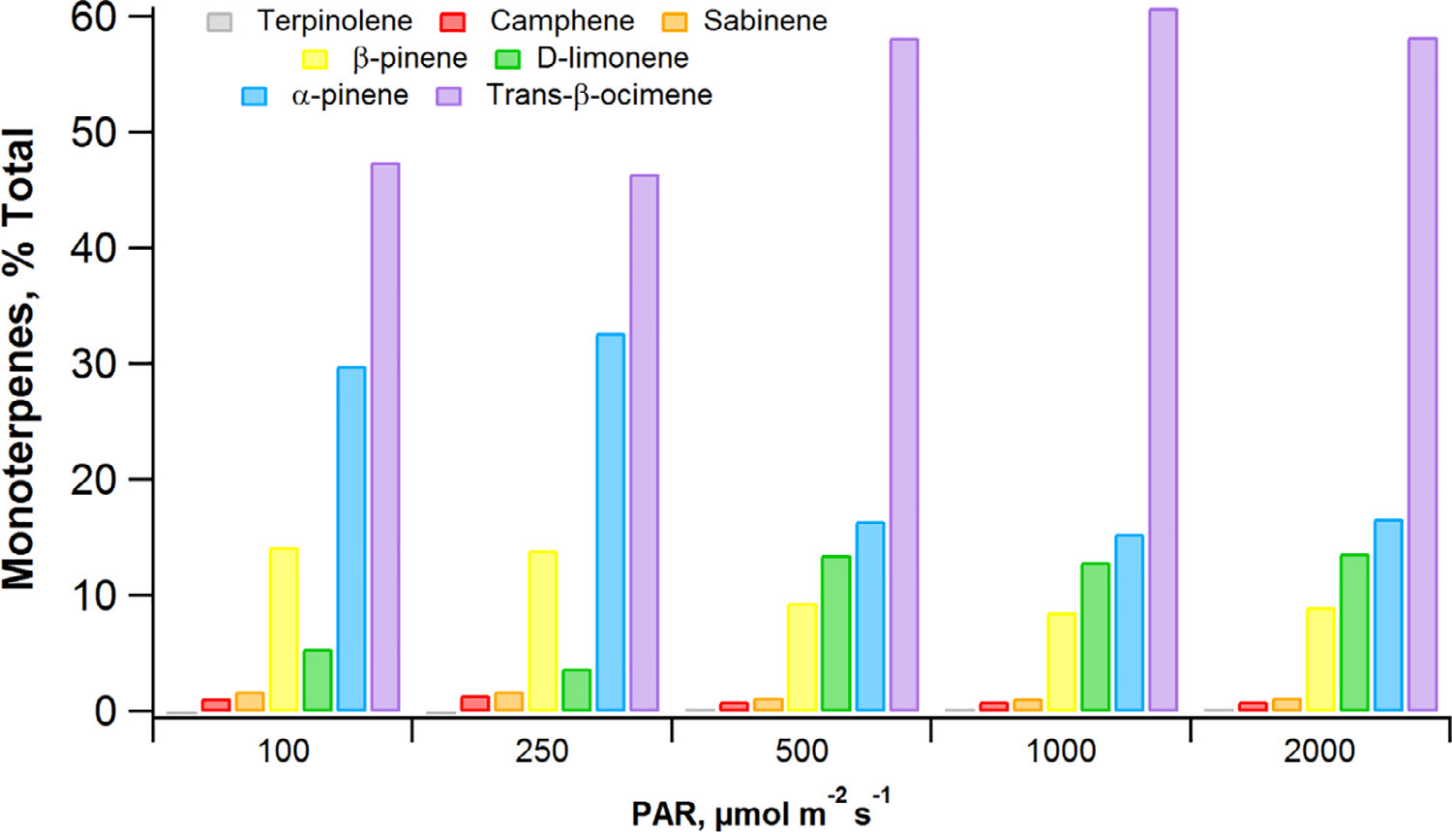
Plot showing the relative abundance (% total) of seven individual monoterpene emissions from a banana leaf as a function of PAR intensity. Note that despite the strong light stimulation of their absolute emission rates, the consistency of the composition of monoterpene emission patterns across PAR levels.

Of the monoterpenes emitted by the banana leaf, at least seven were detected and individually quantified including trans-β-ocimene, α-pinene, d-limonene, β-pinene, camphene, sabinene, and terpinolene. Fig. 3 shows the emission of the four most abundant monoterpenes from the banana leaf with emissions at 2000 µmol m^−2^ s^−1^ PAR of 16.0 nmol m^−2^ s^−1^ (trans-β-ocimene), 4.57 nmol m^−2^ s^−1^ (α-pinene), 3.75 nmol m^−2^ s^−1^ (D-limonene), and 2.49 nmol m^−2^ s^−1^ (β-pinene). While the other three monoterpenes (camphene, sabinene, and terpinolene) showed light dependent emissions, their emission rates were low (< 1.0 nmol m^−2^ s^−1^). Thus, monoterpene emissions from Banana was dominated by trans-β-ocimene. This compound is of particular interest for atmospheric chemistry due to its high reactivity to atmospheric oxidants like ozone and the hydroxyl radical. When the relative abundance of monoterpenes (% total) emitted from the banana leaf was plotted versus PAR intensities, the composition of monoterpenes was found to be largely constant (Fig. 4). Thus, although the absolute emission rates of the monoterpenes dramatically increased with PAR, the relative emissions (% total) were very stable, suggesting similar enzymatic mechanisms for their biosynthesis linked with photosynthesis.

## Conclusions

Despite the fact that global biogenic VOC emissions are known to be dominated by tropical ecosystems, a scarcity of data exists at the leaf level linking compound specific VOC emissions with photosynthesis, transpiration, and stomatal conductance. In this MethodsX paper, we present a new technique for field observations of leaf physiology measurements under controlled environmental conditions coupled with compound specific VOC emissions in remote tropical forests. Beyond the demonstration of active leaf physiology, the critical importance of collecting both leaf photosynthesis and VOC emission observations is highlighted by studies that demonstrate tropical isoprene and monoterpene leaf emission are dependent on recent photosynthesis for both their carbon/energy (ATP/ NADPH) requirements during biosynthesis. Thus, these coupled leaf observations will be critical for the accurate development of global VOC emission models linked with terrestrial photosynthesis. We present an example data-set collected by the portable field system by characterizing the response of seven monoterpenes emissions and net photosynthesis to light intensities from a banana leaf in the central Amazon. The developed system is expected to be widely used in the biological and atmospheric communities studying VOC emissions linked with leaf physiology in the hyper-diverse tropical ecosystems, especially owing to its portable and automated nature and the low detection limit for VOC concentration quantification (10 s of ppt); an important property needed for dynamic leaf enclosure systems in the field. We show that the system is deployable to remote tropical locations including the canopy level on flux towers and fully programmable allowing for automatic collection of VOC emission time series of specific VOCs and photosynthesis in response to environment changes, such as light. We anticipate that future tropical research projects will take advantage of the presented methods, which aim to advance our understanding of the terrestrial carbon cycle and its feedbacks and interactions with the atmosphere (graphical abstract).

## Declaration of Competing Interest

The authors declare that they have no known competing financial interests or personal relationships that could have appeared to influence the work reported in this paper.
